# Early outcomes of patient-prosthesis mismatch following aortic valve replacement

**DOI:** 10.1177/02676591211023286

**Published:** 2021-06-03

**Authors:** Serik Aitaliyev, Egle Rumbinaitė, Karolina Mėlinytė-Ankudavičė, Rokas Nekrošius, Vytenis Keturakis, Rimantas Benetis

**Affiliations:** 1 Department of Cardiac, Thoracic and Vascular Surgery, Hospital of Lithuanian University of Health Sciences Kauno Klinikos, Medical Academy, Lithuanian University of Health Sciences, Kaunas, Lithuania; 2 Department of Cardiology, Medical Academy, Lithuanian University of Health Sciences, Kaunas, Lithuania

**Keywords:** aortic valve, valve hemodynamic, AVR, results, PPM, heart valve prosthesis

## Abstract

**Introduction:**

Patient-prosthesis mismatch (PPM) has been associated with numerous short- and long-term adverse events. This study aimed to evaluate the effect of PPM on early postoperative results after aortic valve replacement (AVR) in daily practice.

**Methods:**

In this single-centre retrospective study, 150 non-consecutive patients from March 2019 to January 2020 with clinically indicated AVR with/without concomitant surgery were analysed. The study protocol included operative mortality, complication rate, and pre- and postoperative echocardiographic data. PPM was considered severe with indexed effective orifice area at <0.65 cm^2^/m^2^, moderate at 0.65–0.85 cm^2^/m^2^ and none at >0.85 cm^2^/m^2^.

**Results:**

Moderate PPM was observed in 16 patients (10.6%). No patient had severe PPM. PPM was not related to early mortality (*r* = 0.40, p = 0.630), intra- (*r* = −0.076, p = 0.352) and postoperative (*r* = −0.0134, p = 0.102) events.

**Conclusion:**

In this study, moderate PPM was a frequent finding after AVR, whereas severe PPM was not observed. PPM did not affect the early results after AVR. A long-term follow-up study in a large patient population is required to assess the actual influence of residual PPM.

## Introduction

Since the recognition of patient-prosthesis mismatch (PPM) by Rahimtoola in the late 1970s, heart specialists have paid critical attention to the effect of PPM on the outcomes after aortic valve replacement (AVR) surgery. The influences of PPM on short- and long-term mortality and morbidity remain controversial.^1–7^ Several determinants of PPM after AVR have been described. Some authors have reported preoperative status, age, and left ventricular (LV) function as the predisposing factors of PPM,^8,9^ while others have suggested using a prosthesis with a low gradient and larger effective orifice area (EOA) to avoid PPM.^10–12^

The incidence of moderate PPM varies between 27.9 and 71% among AVR patients,^9,13–16^ while that of severe PPM ranges between 11 and 22.8%.^8,15,16^ Investigations on the adverse effects of PPM on mortality and morbidity are plenty^1,15,17^ and still ongoing. Studies have reported that PPM has no or minimal effect on majority of patients.^1,16,18^ However, these results did not rule out the dependence of PPM on surgeons’ experience. Besides, Tully et al. reported that AVR and concomitant coronary artery bypass grafting (CABG) surgery contributed to high mortality in moderate and severe PPM patients.^19^

This study aimed to investigate the effects of PPM on early postoperative results after AVR in the daily clinical practice of a university hospital.

## Methods

### Study population

In this single-centre retrospective study, symptomatic patients with moderate or severe aortic stenosis, aortic regurgitation, or mixed aortic valve (AV) dysfunction were enrolled. We analysed 150 patients from March 2019 to January 2020 who underwent AVR with/without concomitant surgery. We divided the study patients into two groups: PPM-free and PPM groups. Patients with previous open-heart surgeries or active endocarditis, and emergency cases were excluded from the study. Moreover, patients who required the aortic root enlargement procedure were excluded from the study. Preoperative and early postoperative data included demographic data, medical history, physical examination, New York Heart Association (NYHA) status, EuroSCORE II, Society of Thoracic Surgeons (STS) score, transthoracic echocardiography and intraoperative transoesophageal data. Additional information, including, the surgical technique and size, type and manufacturer of the valve used was obtained. Preoperative characteristics are given in Table 1.

**Table 1.** Preoperative characteristics.

Variables	PPM free	PPM	*p*-Value
Age (years)	65.71 ± 10.04	63.33 ± 11.58	NS
Female sex	53 (39.5%)	7 (43.7%)	NS
BSA (m^2^)	1.96 ± 0.24	1.89 ± 0.23	NS
BMI (kg/m^2^)	28.59 ± 4.91	27.33 ± 5.51	NS
NYHA class	1.47 ± 0.53	1.5 ± 0.63	<0.05
IV	2 (1.5%)	No	
III	67 (50%)	9 (56.3%)	
II	65 (48.5%)	6 (37.5%)	
I	No	1 (6.3%)	
Low EF	9 (7.7%)	No	NS
Smoker	8 (5.9%)	No	NS
AH	111 (82.8%)	12 (75%)	NS
Dyslipidaemia	82 (61.2%)	9 (56.3%)	NS
Chronic obstructive pulmonary disease	12 (8.9%)	No	NS
Peripheral vascular diseases	26 (19.4%)	3 (18.7%)	NS
DM	19 (14.2%)	1 (6.3%)	NS
History of stroke	7 (5.2%)	No	NS
IHD	84 (62.7%)	9 (56.3%)	NS
MI	25 (18.6%)	No	NS
Renal, liver failure	30 (22.4%)	2 (12.5%)	NS
BAV	42 (31.4%)	5 (31.3%)	NS
Degenerative valve aetiology	127 (94.7%)	15 (93.7%)	NS
STS score (%)	2.04 (0.47–23.50)	1.27 (0.38–9.67)	NS
EuroSCORE II (%)	2.75 (0.50–42.40)	2.5 (0.56–9.0)	NS

BMI: body mass index; BSA: body surface area; NYHA: New York Heart Association; HFpEF: heart failure with preserved LV ejection fraction; AH: arterial hypertension; DM: diabetes mellitus; IHD: ischaemic heart disease; MI: myocardial infarction; BAV: bicuspid aortic valve; NS: non-significant.

All human sections were acquired from the university hospital of the Lithuanian University of Health Sciences. The Regional Medical Research Ethics Committee of the Lithuanian University of Health approved the research protocol Sciences (No. BE-2-69, 17 September 2019). Written informed consent was obtained from all patients.

### Surgical techniques

All patients underwent AVR with/without concomitant surgery using standard cardiopulmonary bypass (CPB) via median (*n* = 147) or three patients via partial upper sternotomy under moderate hypothermia (*n* = 146). Four patients underwent dilated or/and calcified ascending aorta replacement under hypothermic circulatory arrest. The decision to use a biological (tissue) or mechanical valve prosthesis and selection of the valve was left to the preference of the surgeons and patients. In addition, all patients were analysed according to the complexity of the operation on the AVR, AVR ± CABG ± other surgery, and AVR ± other surgery groups.

### Echocardiographic analysis

The following LV measurements and AV parameters were obtained from all patients: LV end-diastolic diameter (LVEDD), LV septal and posterior thickness, LV mass, aortic annulus, sinus of Valsalva, sinotubular junction and proximal ascending aorta. LV ejection fraction (EF) was determined by using the Simpson biplane method. Low EF refers to the LV EF reduced to less than 30%. Left ventricular hypertrophy (LVH) was defined by linear measurements, 95 g/m^2^ in women and 115 g/m^2^ in men.^20^

Echocardiography data, such as peak and mean transprosthetic pressure gradients (G max, G mean), velocity (V), the effective orifice area (EOA) and indexed iEOA (iEOA) were derived at baseline and before discharge. EOA and iEOA were assessed using the continuity equation of velocity-time integral. iEOA refers to EOA divided by the body surface area (BSA). Moreover, projected iEOA was retrieved from the EOA table provided by the manufacturer.^21^ PPM was considered severe at projected iEOA <0.65 cm^2^/m^2^, moderate at 0.65–0.85 cm^2^/m^2^ and no PPM at >0.85 cm^2^/m^2^.

All Doppler measurements were averaged during sinus rhythm for three and five cardiac cycles – with rhythm disturbance. One of the three highly trained sonographers used Philips EPIQ 7G or Philips CX50 (Philips Healthcare, Andover, MA) for transthoracic imaging. For each case, two-dimensional images and colour-flow Doppler in multiple views were captured.

### Data analysis

All normally distributed data are expressed as means ± standard deviations or numbers (percentages). Continuous data with non-normal distributions are represented as median (interquartile range). Differences between continuous variables were tested using Student’s *t*-test or Mann–Whitney test, depending on whether the data were normally distributed or not. Differences between categorical variables were evaluated using chi-squared or Fisher’s exact tests. Relations between variables were analysed through Pearson’s correlation coefficients. The difference was considered significant when the *p*-value was less than 0.05. All statistical analyses was performed using the IBM SPSS Statistics for Windows version 26.0 (IBM Corp., Armonk, NY, USA).

## Results

### PPM rate

Valve types and sizes used in the PPM-free and PPM groups are given in Table 2. More than 70% of the implanted valves were St. Jude Trifecta (St. Jude Medical, Inc., St. Paul, MN, USA). Other valves used included St. Jude Regent (*n* = 16, 10.6%), Sorin Carbomedics (Sorin Group USA Inc., Arvada, CO; *n* = 14, 9.3%), St. Jude Epic (*n* = 4, 2.6%), Medtronic Freestyle (Medtronic Inc., Minneapolis, MN; *n* = 3, 2%), St. Jude Master (*n* = 2, 1.3%), Medtronic ATS (*n* = 1, 0.6%) and St. Jude Master Aortic Valved Graft (*n* = 1, 0.6%).

**Table 2.** Aortic valve prostheses: types and sizes used in the PPM-free and PPM groups.

Prosthesis label and size (PPM/PPM-free)	19	21	23	25	27	29	Overall
Stented tissue valve							
St Jude Trifecta	No/1	No/9	1/29	1/33	No/35	–	2/107
St. Jude Epic	–	–	4/No	–	–	–	4/No
Stentless tissue valve							
Medtronic freestyle	–	–	–	–	No/2	No/1	No/3
Mechanical valve							
St. Jude Regent	–	–	–	No/8	No/8	–	No/16
Sorin Carbomedics	–	1/No	5/1	4/1	No/2	–	10/4
St. Jude Master	–	–	–	No/1	No/1	–	No/2
Medtronic ATS	–	–	No/1	–	–	–	No/1
St. Jude Master Aortic Valved Graft	–	–	–	–	No/1	–	No/1

In the present study, manufacturer provided EOA was used to determine the iEOA and PPM rate. Moderate PPM occurred in 16 (10.6%) patients at the time of discharge. No severe PPM was observed during the study. However, moderate PPM was observed with 21, 23, 25-mm Sorin Carbomedics prostheses in 1, 5 and 4 patients, respectively. Four PPM patients had 23-mm St. Jude Epic valve, and isolated incidents were observed with 23 and 25-mm St. Jude Trifecta valves.

There was a clear relation between PPM and the prosthesis size (*r* = −0.236, *p* < 0.05, *N* = 150) and type by prosthesis manufacturer (*r* = 0.384, p < 0.001, *N* = 150), in our study. Moreover, PPM correlated with the prostheses groups, namely tissue and mechanical (*r* = 0.329, p < 0.001, *N* = 150). However, no relationship between projected and in vivo iEOA was found (*r* = 0.192, p = 0.94, *N* = 77).

### Subject characteristics

Preoperative patient characteristics were comparable between the PPM and PPM-free groups. The PPM group had a higher mean NYHA class than the PPM-free group (*p* < 0.05). Nine (56.3%) patients in the PPM group and 84 (62.7%) in the PPM-free group experienced ischaemic heart disease, but the difference between the groups was not statistically significant. Comorbidities, such as arterial hypertension (AH), dyslipidaemia, chronic obstructive pulmonary disease (COPD), peripheral vessel diseases, diabetes mellitus (DM), myocardial infarction (MI), renal and liver failure prevailed in the PPM-free group compared to the PPM group; however, the differences were not significant.

### Surgical techniques

Operative data are presented in Table 3. Majority of the valves (96.7%) were implanted at the intra-annular positions with continuous running 2-0 Prolene sutures and 3.3% of the valves were implanted at the supra-annular positions using interrupted pledgeted mattress sutures. To note, bioprosthesis more frequently implanted in the PPM-free groups, whereas mechanical prosthesis were used in the PPM group (*p* < 0.001). Mean prosthesis size was bigger in the PPM-free group compare to those in the PPM group (*p* < 0.05).

**Table 3.** Operative data.

Variables	PPM-free	PPM	p Value
Prosthesis size, mean ± SD	24.99 ± 1.97	23.50 ± 1.15	<0.05
Bioprosthesis, *n* (%)	110 (82.1)	6 (37.5)	<0.001
Mechanical prosthesis, *n* (%)	24 (17.9)	10 (62.5)	<0.001
AVR, *n* (%)	43 (32.1)	4 (25)	NS
AVR ± CABG ± other surgery, *n* (%)	71 (52.9)	9 (56.3)	NS
AVR ± other surgery, *n* (%)	20 (14.9)	3 (18.7)	NS
CPB time (min), mean ± SD	111.44 ± 43.97	121.13 ± 34.11	NS
Cross-clamp time (min), mean ± SD	70.10 ± 26.24	77.56 ± 25.36	NS
Operation time (min), mean ± SD	212.39 ± 65.39	220.00 ± 35.39	NS
Length of ICU stay (days), *n* (range)	4.00 (1–49)	4.00 (1–35)	NS
Length of postoperative stay (days), *n* (range)	12.00 (1–98)	11.50 (7–129)	NS

CPB: cardio-pulmonary bypass; ICU: intensive care unit; NS: non-significant; SD: standard deviation; n: number.

Four patients underwent AVR under circulatory arrest for 12.75 ± 4.9 (9–20) minutes. No differences in CPB time, cross-clamp time, operation duration, intensive care unit (ICU) and postoperative stay were found comparing both groups.

Distribution and frequency of AVR, AVR + CABG + other surgery and AVR + other surgery in both groups were similar. Despite the fact that all deceased patients had undergone AVR + CABG ± other surgery, mortality did not correlate with the type of surgery (*r* = 0.055, *p* = 0.508, *N* = 150).

### Postoperative mortality and valve-related complications

The details of the early clinical events are given in the Table 4. There were six hospital deaths (4%): five (3.7%) cases in the PPM free and one (6.3%) case in PPM groups. One patient died from defibrillation-resistant ventricular arrhythmia on the same day of surgery, four patients died from postoperative respiratory failure and one from postoperative respiratory failure and congestive heart failure three weeks post operation. There were no statistical differences in the mortality rates between the PPM-free and PPM groups.

**Table 4.** Early outcomes.

Outcomes (*n* = 150)	PPM free (%)	PPM	*p*-Value
Early mortality	5 (3.7)	1 (6.3%)	NS
Intraoperative complications	7 (5.2)	No	NS
Low cardiac output syndrome	3 (2.3)	No	NS
De novo AR	2 (1.5)	No	NS
Systolic anterior motion (SAM)	1 (0.7)	No	NS
Aortic hematoma	1 (0.7)	No	NS
Postoperative complications	71 (52.9)	5 (31.3%)	NS
AF	29 (21.6)	1 (6.3%)	NS
Postoperative respiratory failure	13 (9.7)	No	NS
Delirium	12 (8.9)	1 (6.3%)	NS
Sternal infection	6 (4.5)	No	NS
Prolonged ventilation	17 (12.7)	3 (18.7%)	NS
Cardiogenic shock	5 (3.7)	No	NS
Pacemaker	4 (2.9)	No	NS
New-onset dialysis	5 (3.7)	No	NS
Early stroke	3 (2.3)	No	NS
GI bleeding	3 (2.3)	1 (6.3%)	NS
Tamponade	2 (1.5)	1 (6.3%)	NS
AV block I type	3 (2.3)	No	NS
AV block II type	1 (0.7)	No	NS
AV block III type	2 (1.5)	No	NS
TIA	2 (1.5)	No	NS
Urinary infection	2 (1.5)	No	NS
Liver insufficiency	2 (1.5)	No	NS
Bleeding	2 (1.5)	No	NS
Respiratory distress syndrome	2 (1.5)	No	NS

AR: aortic regurgitation; AF: atrial fibrillation; GI bleeding: gastrointestinal bleeding; AV block: atrioventricular block; TIA: transient ischaemic attack; NS: non-significant.

Complications were divided into intraoperative and early postoperative complications (from the second postoperative day to the day before discharge). Despite the fact that intraoperative and postoperative events frequently occured in the PMM-free group, rather than in the PPM group, there was no statistical difference between the two groups. Moreover, there is no relations between PPM and early mortality (*r* = 0.40, p = 0.630, *N* = 150), and intra- (*r* = −0.076, p = 0.352, *N* = 150), and postoperative (*r* = −0.0134, p = 0.102, *N* = 150) events.

### Hemodynamic performance

No significant differences were found in the echocardiographic data of LV parameters and functions preoperatively and at discharge between the groups (Table 5). However, hemodynamics was at discharge significantly different between the two groups. V max, G max and G mean were higher in the PPM than in the PPM-free group (V max: 2.59 ± 0.56 m/s vs 1.84±0.37 m/s, *p* < 0.001; G max: 29.83 ± 9.29 mmHg vs 14.37 ± 6.08 mmHg, *p* < 0.001; G mean: 15.24 ± 7.16 mmHg vs 7.48 ± 3.45 mmHg, *p* < 0.001). Moreover, EOA, iEOA and projected iEOAof the PPM group were significantly smaller than those in the PPM-free group (*p* < 0.05). Although the preoperative aortic annulus was not statistically different between the two groups, the aortic annular size at discharge was smaller in the PPM group than that in the PPM-free group (*p* < 0.05).

**Table 5.** Preoperative and postoperative echocardiography data.

Variable	PPM-free	PPM	p-Value
Preoperative			
LVEDD (mm)	50.79 ± 7.66	47.46 ± 6.28	NS
iLVEDD (mm/m^2^)	26.42 ± 3.93	25.09 ± 3.02	NS
LV septal thickness (mm)	14.01 ± 3.79	13.87 ± 2.46	NS
Posterior wall thickness (mm)	12.05 ± 1.81	12.13 ± 1.32	NS
LV mass (g)	267.86 ± 61.51	244.33 ± 70.17	NS
iLV mass (g/m^2^)	124.07 ± 27.48	111.18 ± 26.31	NS
LVH	92 (68.6%)	11 (68.7%)	NS
LV EF (%)	48.64 ± 10.48	52.78 ± 6.08	NS
Aortic annulus (mm)	23.97 ± 2.69	22.89 ± 1.74	NS
V max (m/s)	4.09 ± 1.18	4.51 ± 0.79	NS
G max (mmHg)	72.61 ± 38.29	84.06 ± 29.25	NS
G mean (mmHg)	46.05 ± 21.47	48.93 ± 17.68	NS
EOA (cm^2^)	0.96 ± 0.51	0.88 ± 0.15	NS
iEOA (cm/cm^2^)	0.49 ± 0.16	0.51 ± 0.19	NS
Postoperative (at discharge)			
LVEDD (mm)	48.83 ± 6.16	47.40 ± 5.83	NS
iLVEDD (mm/m^2^)	25.06 ± 3.19	24.96 ± 3.02	NS
LV septal thickness (mm)	13.08 ± 1.88	12.13 ± 2.06	NS
Posterior wall thickness (mm)	12.03 ± 1.56	11.79 ± 1.42	NS
LV mass (g)	244.32 ± 59.89	211.94 ± 58.79	NS
iLV mass (g/m^2^)	124.07 ± 27.48	111.18 ± 26.32	NS
LV EF (%)	46.24 ± 8.08	50.33 ± 5.16	NS
Aortic annulus (mm)	24.85 ± 2.25	23.10 ± 1.82	>0.05
V max (m/s)	1.84 ± 0.37	2.59 ± 0.56	>0.001
G max (mmHg)	14.37 ± 6.08	29.83 ± 9.29	>0.001
G mean (mmHg)	7.48 ± 3.45	15.24 ± 7.16	>0.001
EOA (cm^2^)	2.83 ± 0.79	1.94 ± 0.64	>0.05
iEOA (cm/cm^2^)	1.43 ± 0.37	1.02 ± 0.34	>0.001
Projected iEOA (cm/cm^2^)	1.12 ± 0.19	0.77 ± 0.05	>0.05

EOA: efficient orifice area; G: gradient; iEOA: indexed efficient orifice area; iLV mass: indexed left ventricular mass; iLVEDD: indexed left ventricular end-diastolic diameter; LF/LG: low flow/low gradient; LVEF: left ventricular ejection fraction; LV mass: left ventricular mass; LVEDD: left ventricular end-diastolic diameter; V: velocity.

## Discussion

This study describes the ‘real-world’ incidence of PPM in patients with different clinicopathological features. We conducted a retrospective study, using data from an open-heart surgery centre with an annual volume of 1000 patients, in a large cohort of AV disease patients to determine the effect of PPM on early postoperative outcomes. We did not restrict our research to patients’ age, valve pathology, surgery type and prosthesis selection. We also analysed unselected data from the general Lithuanian population.

Controversial data exist regarding the impact of PPM on AVR outcomes. A recent meta-analysis showed a significant influence of PPM on both short- and long-term mortalities.^22^ In our study, no obvious differences were detected regarding intraoperative and postoperative complications between the PPM and PPM-free groups. A previous retrospective study demonstrated a clear relationship between severe/moderate PPM and short-/mid-term mortality.^23^ Other studies also showed detrimental effects of PPM on both short- and long-term mortalities.^3,24^ Furthermore, severe PPM,^25^ not moderate PPM, reportedly influenced early and mid-term survival.^26^ Some of the predictors of mortality reported in the previous studies included age, LV dysfunction, bypass time, EuroSCORE II and diabetes.^4,25,26^

Although the rate of moderate PPM was 54% in a recent study,^15^ it was only 10.6% in our study. In our previous paper, we detected a PPM rate of 9.5% using the continuity equation.^27^ We speculate that the low PPM rate might be related to the excellent hemodynamic conditions of the St. Jude Trifecta (>70%) in our study. Several studies presented favourable hemodynamics with St. Jude Trifecta in the AV position and considered it as the best option for AVR surgery.^28,29^ Moreover, resting and exercise hemodynamics of the stented St. Jude Trifecta were similar to that of the stentless Medtronic Freestyle valve.^30^ In a systematic review and meta-analysis by Phan et al.,^31^ attention was focused on the favourable mean gradient and EOA of this valve. Another reason for the low PPM rate was the surgical technique that employed meticulous debridement of the aortic annulus and the predominant use of continuous running suture technique that showed bigger annulus postoperatively comparing to the one measured preoperatively. Probably, Ethibond pledgeted sutures can be obstacles for proper hemodynamics in the prosthesis. A recent study examined the influence of three different suture techniques on PPM.^32^ Authors concluded that the non-pledgeted suture technique reduced PPM rate in small aortic annulus patients. Therefore, PPM incidence has declined over time due to the awareness of its negative consequences and the availability of suitable valve alternatives.^15^ Clearly, most PPM cases are dependent on the surgeon and can be avoided at the time of surgery.

The Carbomedics heart valve is a widely used mechanical prosthesis in our institution. Despite the fact that the majority of PPM cases in our study were observed with the Carbomedics prosthesis, many studies reported excellent hemodynamics.^33,34^ However, several in vivo and in vitro studies showed the discrepancy between the prosthesis size and the hemodynamic profile of the Carbomedics prosthesis.^35,36^ Authors found a weak inverse correlation between prosthesis size and transprosthetic gradients and EOA. Probably, overestimation of Doppler examination and underestimation of EOA by the manufacturer may be the reasons for the discrepancy between clinical and instrumental data.

Our data cannot fully support the finding of a meta-analysis that identified old age and coronary artery disease as the risk factors for PPM.^19^ The mean prosthesis size was different between the PPM and PPM-free groups and they strongly correlate with the PPM rate. In our study, the small prosthesis (19 or 21 mm) was not used in the PPM group. A recent study suggested that not only the small aortic annulus, but also a larger BSA, LVH, tissue valve prosthesis, old age and systemic hypertension, were the risk factors for PPM.^37^

Many studies have suggested the following steps for avoiding PPM.^38–41^ Firstly, the patients’ BSA should be calculated to find the valve with a greater projected EOA. If other valves are not available, mechanical prosthesis or an aortic homograft should be considered. While attempts to implant a big prosthesis in a small aortic annulus (or prosthesis oversizing) deteriorates prosthesis hemodynamics,^42^ aortic root enlargement can facilitate the implantation of valves of a suitable size. Secondly, an unavoidable PPM case, should be accepted if a patient follow a sedentary lifestyle without LV dysfunction. In our study, the manufacturer provided an EOA-predicted PPM rate, which could be used preoperatively.

There are some limitations to the study. The sample size was small and population is heterogeneous. Because of the small PPM rate, we were unable to detect more factors that could affect the PPM occurrence. Furthermore, we could not obtain detailed echocardiographic data of the deceased patients. Concomitant procedures also can limit our study. Studies with long-term follow-ups are required to assess the actual influence of residual PPM on a large population.

## Conclusion

In this study, moderate PPM was frequently found after AVR, whereas severe PPM was not observed. PPM did not affect the early results after AVR. A long-term follow-up study on a large cohort is required to assess the actual influence of residual PPM.
